# Rhythm and timing in autism: learning to dance

**DOI:** 10.3389/fnint.2013.00027

**Published:** 2013-04-19

**Authors:** Pat Amos

**Affiliations:** Training and Private ConsultationArdmore, PA, USA

**Keywords:** autism, cross-modal perception, movement, rhythm, sensory perception, synchrony, timing

## Abstract

In recent years, a significant body of research has focused on challenges to neural connectivity as a key to understanding autism. In contrast to attempts to identify a single static, primarily brain-based deficit, children and adults diagnosed with autism are increasingly perceived as out of sync with their internal and external environments in dynamic ways that must also involve operations of the peripheral nervous systems. The noisiness that seems to occur in both directions of neural flow may help explain challenges to movement and sensing, and ultimately to entrainment with circadian rhythms and social interactions across the autism spectrum, profound differences in the rhythm and timing of movement have been tracked to infancy. Difficulties with self-synchrony inhibit praxis, and can disrupt the “dance of relationship” through which caregiver and child build meaning. Different sensory aspects of a situation may fail to match up; ultimately, intentions and actions themselves may be uncoupled. This uncoupling may help explain the expressions of alienation from the actions of one's body which recur in the autobiographical autism literature. Multi-modal/cross-modal coordination of different types of sensory information into coherent events may be difficult to achieve because amodal properties (e.g., rhythm and tempo) that help unite perceptions are unreliable. One question posed to the connectivity research concerns the role of rhythm and timing in this operation, and whether these can be mobilized to reduce overload and enhance performance. A case is made for developmental research addressing how people with autism actively explore and make sense of their environments. The parent/author recommends investigating approaches such as scaffolding interactions via rhythm, following the person's lead, slowing the pace, discriminating between intentional communication and “stray” motor patterns, and organizing information through one sensory mode at a time.

“…movement must itself be considered a perceptual system.”

(Thelen and Smith, 1994, p. 193; emphasis in original)

Everyday descriptions of social interaction are rich in figures of speech that derive from rhythm and timing in general, and dance or music in particular. If we are in love, we may describe the feeling as “two hearts beating as one,” being “swept off our feet,” feeling “in the groove,” or experiencing “good vibes.” We joke that “it takes two to tango,” and may patronize a well-known online dating site that sums up its promise as “harmony.” Encountering socially maladroit individuals, we describe them as having two left feet, being out of step, being off beat, or stepping on our toes. “Timing,” we declare, “is everything.”

Yet we have the capacity to empathize with people who move differently, and have popularized many affirmations based on Thoreau's advice to those who hear “a different drummer.” Researchers and therapists seem drawn to these images as well, characterizing developmentally vital interactions between parent and child as a dance of relationship and writing about how to support children who fall out of synch in that dance (Fogel, 1993; Maurer, 1994, 1996; Stern, 2000; Wieder and Greenspan, 2005; Trevarthen, 2011). Is it possible that the choice of these terms is far more than a literary flourish, embodying something intuitive and essential about how human beings relate? Could a closer examination of what is happening when people on the autism spectrum seem to move to a different drummer encourage breakthroughs in how we partner with them, and create better vibes all around? As a parent, that would be music to my ears.

It has been my privilege to observe the development of my own children (and others with whom I have worked) at a micro level over many years, and to have emerged with skepticism about the prevailing narrative which portrays people with autism as essentially aimless, unmotivated carriers of static deficits or traits. When my children were very young, it became clear that the typical diagnostic process did not recognize the limitations of its “snapshot viewed from afar,” and that the field was neither disposed nor equipped to notice the dynamic adaptations of which parents become acutely aware. In particular, I became impressed by the ways in which autism, explained in the literature as a brain-based challenge to cognition, in fact presented as deeply embodied: my children appeared to be constantly negotiating with their bodies via strategies that looked quite complicated, were very dependent on task conditions, yielded highly variable results, disintegrated in demand situations, and sometimes looked startlingly similar to the struggles with neurodegenerative conditions experienced by older family members. It surprised me that sustaining a dance of relationship could take so much concentration. When my oldest son, around age six, started to search his body for imaginary buttons, pressing them hopefully to make it function, the metaphorical light bulb turned on: we needed an approach to autism that would recognize and join him in exploring movement and perception. We still do, but are getting closer. This review will trace some converging lines of enquiry.

## Nervous systems and connectivity

The flow of information in our bodies involves two basic sets of systems: the central nervous systems or CNS (brain and spinal cord) and the peripheral nervous systems (composed of the sensory nervous system, which sends information to the CNS from the external environment and internal organs, and the motor nervous system which sends information from the CNS to muscles, glands, and organs, including the skin). As information is constantly adjusted through an ongoing stream of internal and external motion, it moves in two directional flows: from the central to the peripheral nervous systems, and from the peripheral to the central nervous systems. Challenges to bodily rhythm and timing can take a variety of forms depending on which of these flows is involved, and also on whether the “beat of a different drummer” is emerging from involuntary or automatic responses of the nervous systems (e.g., the fight or flight responses of fear, reflexes) or from a lack of voluntary control (e.g., paralysis, dyspraxia, the results of illness or disease).

Dangerous and dramatic central to peripheral disruptions of brain and body rhythm may occur when the usual periodicities of brain waves suddenly veer into chaotic states, resulting in the seizures experienced by many people with autism. Although the comorbidity rate is not well-defined, the Autism Society (2012) estimates that somewhere between 11 to 39% of people with autism develop seizures; published research cites rates as high as 40% (Gabis et al., 2005). In comparison, the prevalence rate for active epilepsy in the general population is between 0.4% and 1% (World Health Organization, 2009). A recent EEG study of children with autism indicated that over 85% had abnormal brain-wave patterns, even if they did not result in overt seizures (Yasuhara, 2010). Non-convulsive seizure activity may manifest as changes in affect and behavior, including dissociative experiences and altered perceptions of body and environment.

These types of central to peripheral disruptions of bodily rhythms are relatively easy to diagnose, and have received a large share of research attention. But human lives are shaped and defined by many other kinds of rhythms, often involving the peripheral to central flow of information. Among people diagnosed with autism spectrum disorders (ASDs), difficulties with sensory modulation (often visible as hyper- or hypo-reactivity) and with voluntary motor activity (Donnellan et al., 2013), as well as unusual registration and/or expression of pain—an understudied area due to difficulties in identifying pain behavior (Nader et al., 2004)—may indicate challenges to the functioning of the afferent somatic systems, while frequent reports of unusual diet and gastrointestinal problems may indicate dysregulation of the enteric systems (Horvath and Perman, 2002). These challenges affect not only the efficiency of peripheral flows of information within the body, but the functioning of those predictably-timed cyclic flows between the natural (and, ultimately, the social) environment and peripheral nervous systems which are known as circadian rhythms.

The circadian rhythms give us daily cycles of sleep and wakefulness, hunger, body temperature, and brain waves, as well as hormonal peaks and ebbs. Associated with these short-term natural rhythms may be fluctuating states of sensory arousal and alertness. People diagnosed with an ASD often appear to experience states that fluctuate more widely and more often, sending them out of sync with typical daily rhythms. Parents may report that their offspring experience unpredictable daily cycles including difficulty registering hunger and shortened, sporadic intervals of sleep (Malow, 2004; Hu et al., 2009; Glickman, 2010). One study found sleep disturbances among 52% of subjects with autism vs. 7% of their typically developing siblings (Horvath and Perman, 2002). Evidence has also been found for “clock gene” anomalies in autism, which may affect sleep, memory, and timing (Nicholas et al., 2007).

Many parents and teachers of children with autism also report significant challenges to the regulation of mood and activity that appear linked to seasonal changes, such as the daylight-related Seasonal Affective Disorder with its apt acronym of SAD. In this case we might argue that an overall pattern of behavior *does* predictably track a biological temporal rhythm; unfortunately, unless one is a hibernating mammal it represents an adaptation to the wrong situation at the wrong time-scale; as such it can interfere with reception of the shorter-term behavioral cues to which humans must attend.

Preliminary research into the effects of disruptions of circadian timing suggests that “Behavioral disturbances in ASD may arise in part from an inability of an individual's circadian oscillator to entrain to environmental and social cues” (Barnard and Nolan, 2008, para. 15). When the internal clock cannot reliably, consistently match or mirror the environmental and social rhythms of typical activity, long-term consequences may be profound. The circadian time of brain rhythms and physiological cycles is tightly entwined with developmental time, the engine of which is the time sensitive everyday experiences in which all children engage—and in which children with autism appear to engage in different ways, over different periods, using different strategies.

The “dance of relationship” by which newborn child and caregiver begin to make mutual sense of their world is understood to be based on the co-production, or entrainment, of their actions (Fogel, 1993; Stern, 2000). Clearly we need to know more about how this dance works when the different metronomes of different nervous systems do not readily fall into sync. It seems possible that all of these levels of rhythm and timing, from circadian time to developmental time, and from the real time self-synchrony of perception and movement to the real time interactional synchrony of communication and social activity, might one day be woven into a unified field theory that makes sense of the amazingly diverse ways humans move to the rhythm of time.

## Autism research: a missing piece?

Over the last few decades, a significant body of research has zeroed in on challenges to neural connectivity as key to understanding autism (Belmonte et al., 2004). Previous theories have been laid successively to rest, including the early suggestion that autism may be a traumatic response to cold parenting [Bettelheim, 1967; see response in Rimland (1964)]; an extreme language impairment (see Bartak et al., 1975), a single basic learning impediment such as a limited attention span, an amnesiac disorder, or an auditory processing deficit (see Minshew and Rattan, 1994); or closely correlated with intellectual disability (see Edelson, 2006). Recent interest in the supposed inability of children with autism to invoke a Theory of Mind (ToM) (Baron-Cohen et al., 1985) faltered once this multidimensional concept was deconstructed (see Rogers et al., 2007). Many researchers now argue that no such inborn mental mechanism exists, and look instead to the study of dynamic systems to understand different embodied experiences (Shanker, 2004).

The study of dynamic systems offers autism researchers a way out of the old debate between genetics and environment, the heavyweight determinisms that traditionally stood sentinel over child development studies. To the autism field they brought the dubious gifts of prognosis at the time of diagnosis, and of an aggressive form of behaviorism constructed on an engineering model. Viewed as a dynamic system, however, the development of *any* child can be seen to emerge without a top-down genetic script or one-way environmental chutes and ladders, through ongoing perceptions and actions. Knowledge itself appears as an action-based process (Thelen and Smith, 1994, p. 247). The movement perspective on autism offers evidence of an overabundance of variability in this process of assembling reliable embodiments of knowledge: the process continues, but is overloaded and precarious; the categories generated may increasingly reflect the elaboration and playing out of a different perceptual system. Instead of approaching such children as non-learners, the new research task becomes one of discovering the dynamic adaptations of their individual learning trajectories and intervening to decrease the noisiness that seems to occur in both directions of neural flow. Such research may suggest the neurological basis of a dynamic model to explain the challenges to movement and sensing, and ultimately the differences in developmental trajectories, found across the autism spectrum.

In overviews of decades of autism research, Donnellan (1999), Donnellan and Leary (1995), Donnellan et al. (2010, 2013), and Gowen and Hamilton (2012) identified the exploration of movement differences and their impact on the rhythms of daily life as crucial to a better understanding of the experiences of people on the autism spectrum. Developmental approaches to children with autism recommend “following the child's lead” sympathetically (Greenspan, 1992, 1997; Greenspan and Wieder, 1997, 2006) and emphasize the importance of the dance of relationship; occupational science, with its emphasis on fostering sensory integration and regulation, has become increasingly valued as a source of approaches for enhancing bodily synchrony and *praxis*, the ability to plan actions and engage meaningfully with the world (Williamson et al., 2000). Speech-language pathologists, providers of assistive and alternative communication supports, and various types of music therapists also emphasize the use of rhythm and timing as scaffolding to build social and communicative interactions (Schögler, 2008; Hardy and LaGasse, 2013).

The recognition of movement difficulties, however, has not necessarily led to accurate interpretations of their nature. A persistent belief is that sensory uptake at the level of the primary sensory organs must not function accurately; people with autism are sometimes described as unable to receive basic sensory information from their environment. To the contrary, a significant body of research confirms that the sensory systems function properly at their initial tasks of registering input (Minshew and Rattan, 1994), including the proprioceptive sense of limb position (Fuentes et al., 2011). It is the ability to make reliable, intentional use of this input that appears to malfunction, a finding consistent with the descriptions of self-advocates such as Nick Pentzell:

*To have autism is like having a short in a computer. I know what I want to do, but my body gets confused and it does not correctly carry out the order my brain sends it. I take in information, but my body scrambles the output* (Young, 2011, p. 164).

For such individuals, unusual challenges and exceptional skills can exist side by side, in the same brain domains (Williams et al., 2006). Self-advocate Sue Rubin reflects:

*It is funny how we are considered strange or different, even though our recollection of complex patterns, memory for precise detail, and overall capabilities many times exceed those of the people who are pointing or staring* (Young, 2011, p. 107).

This research does not implicate certain types of information, such as language or social interaction, as inherently too complex; rather, it suggests that something about the ways information is structured or becomes available may overwhelm a highly sensitive processing system (Williams et al., 2006). The whole then becomes less than the sum of its parts.

One question we can put to the connectivity research concerns the role of rhythm and timing in this delicate operation and, in particular, whether they can be mobilized to reduce overload and enhance performance. To respond, researchers will need to move beyond the well-documented connectivity challenges in the cortical regions that dominate current autism research, and recognize that this brain-based model remains partial because it is disembodied. The missing piece may be a consideration of connectivity challenges in the peripheral nervous systems. Replicable, testable theories about what occurs in these systems for children with ASDs have been scarce to non-existent, which may help explain the widespread bias toward envisioning autism as fully rooted in the neural processing of key cortical regions, and the disinclination to attach much significance to sensorimotor phenomena, which tend to be reflected in diagnostic protocols as secondary or optional criteria. In contrast to studying people with autism as if only central cortical structures and connections contribute to development, researchers need to look at movement itself: as sensed and organized, conscious and unconscious, volitional and non-volitional, as it plays out at different levels in the peripheral nervous systems, and as these systems interface with the CNS to develop a dynamic, self-organizing map of the body in space and time.

## Rhythm and timing in early development

It may be helpful to consider how sensory input is organized in typical child development, and then bring to bear some observations of infants who were later diagnosed with autism. Newborn infants capture our attention precisely because they do not respond as “blank slates”; instead, infants quickly begin to organize responses to sensory input, charming caregivers with tightly-timed reflections of their own actions. The development of proprioception and vestibular processing involve them in learning not only how their bodies move in space, but how they move in time. The trajectory of any action involving coordinated movement, from walking through a moving crowd to jumping into a conversation, can only be projected if time is accurately weighed in the equation. Time can also be seen as *emerging* from the equation because time perception is bound up with movement; we talk about approaching “points in time,” “moving through time,” and events being “distant in time” because we sense that movements of the body, both external and internal, are involved in assembling a perception of time's “placement” relative to our trajectory. Like our movements, time is perceived consciously and unconsciously, deliberately and unintentionally; it both flows from bodily motion and flows back to guide its course.

We call the dual-natured, split-second calculation that guides our coordinated movements “timing” and, when it is part of a broader ability to plan and strategize, we refer to a person as having a “sense of time.” Yet a sense of time is not associated with any specific sensory system; it is registered through the peripheral nervous systems as they carry motor and sensory information to and from the CNS. In the CNS, time perception is associated with a highly distributed brain system including the cerebral cortex, cerebellum, and basal ganglia—areas of the brain generally associated with autism (Bauman and Kemper, 2005). As was the case with the connectivity challenges discussed earlier, however, researchers have paid more attention to the function of the cortical regions in time perception than to the peripheral nervous systems. In assuming that the neocortex is the only source of the intelligent forces—timing, decision making, planning—driving development, much of the autism field continues to ignore the ways that intelligence is embodied and may be engaged through a dance of relationship.

Timing seems to operate as the common link that binds sensory experiences into a coherent whole. Infants move in carefully-timed synchrony with caregivers in a dance-like exchange that creates the framework for a child's first experiences of actors, actions, and things acted upon. The importance of the shared information that emerges through this engagement is profound, as child development and autism researcher Colwyn Trevarthen makes clear:

Most impressively, an alert newborn can draw a sympathetic adult into synchronized negotiations of arbitrary action, which can develop in coming weeks and months into a mastery of the rituals and symbols of a germinal culture, long before any words are learned (Trevarthen, 2011, p. 121).

When the timing of these early experiences is “off” it can trigger a cascade of consequences for development (Trevarthen et al., 1998; Greenspan and Shanker, 2007). Many parents of infants who were later diagnosed with autism report that their baby was difficult to engage, following either a pattern of muscle tension and hyperarousal which left them difficult to sooth, or being “floppy” and difficult to arouse; babies may also oscillate between these states (Williamson et al., 2000). However, some parents recall what they felt was a typical infancy, and in most cases the early motor milestones of these babies reportedly are met on time (Centers for Disease Control and Prevention, 2010).

Even families who do not recall particular concerns in the first few months tend to start reporting them around the 12–15 month mark, when the child's preverbal communication appears to lag. As developmental experts point out, this is the time period when social development—manifested through rapid leaps in language, reciprocal interactions, and joint attention—begins to heat up. The beat gets faster, the steps more complex, and the perception of difficulty keeping up with peers is heightened (Greenspan and Shanker, 2007). Somewhere in the second or third year, it becomes obvious that the child needs support in the dance of relationship (Greenspan, 1992). But the question remains: what has been happening in the child's development before social and communication differences became pronounced, and before autism was suspected? Answering that question might lead to an understanding of the dynamics of autism, and help us discover the *kinds* of support needed. We would not need to untangle the complex etiologies of the ASDs or the origins of a particular child's autism; instead, we would need to discover how the child operates in and makes sense of the world, and how the child's experiences are creating—or not creating—a stable and reliable basis from which to extrapolate into new situations and timeframes.

Several lines of investigation may be converging on an intriguing answer to this question of child development, and may deeply implicate the rhythm and timing of sensorimotor experience. They include demonstrations that, even in the first months of life, when those first motor milestones are being met, babies on their way to an autism spectrum diagnosis are meeting them differently (Teitelbaum et al., 1998, 2004). These findings are supported by recent data from a variety of studies using different measures, which suggest that “80–90% of children with ASD show some degree of motor abnormality” (Hilton et al., 2011, p. 4), “with 95% of one sample demonstrating” some degree of sensory processing dysfunction (Tomchek and Dunn, 2007, p. 198). They are underscored by the observations and that “over 90% of children with autism had sensory abnormalities and had sensory symptoms in multiple sensory domains” (Leekam et al., 2007, p. 894), and are underscored by the observations of numerous self-advocates with ASDs.

Unfortunately, the existence of separate studies confirming motor differences and sensory differences also suggests that the lines of investigation are not converging seamlessly: in the autism literature motor differences remain isolated from sensory challenges, a situation which obscures their nature and neurological dynamics. Movement is, as Thelen and Smith insisted in their groundbreaking work on cognition and action, a perceptual system (1994, p. 193); to move is to perceive, and to perceive is to move. Yet the current generation of parents and therapists, drawing on and popularizing the bifurcated research literature, is now glossing children's motor difficulties as “clumsiness” and making a clumsy distinction between “sensory” challenges and “behavior” challenges. The exploration and dissemination of a neurologically-grounded, fully embodied and dynamic developmental model for thinking about movement needs to become a priority.

Starting in the mid-1990's, a team of researchers at the University of Florida began gathering home videos made by the parents of infants and toddlers who were later diagnosed with autism (Teitelbaum et al., 1998) and Asperger syndrome (Teitelbaum et al., 2004). In each case, researchers found differences in the ways these babies used their bodies to interact with their environments. Within the first collection of videos there appeared a number of challenges that continuing research confirmed as characteristic, among them: difficulties in self-synchronizing the body to roll over and to crawl; a lack of superimposed movements; a lack of protective reflexes; failure to exploit allied reflexes to enhance movement; the preservation of motor patterns from an earlier stage of development, as if physical development itself were occurring out of sync; and difficulty coordinating arms and head to explore objects. The researchers suggested that these differences had gone unremarked because the action *does* get performed. The story, they emphasized, is in *how* it gets performed.

Watching the subtle struggles embodied in these videos, viewers are reminded of the ways typically developing children proceed to capture their bodies' spontaneous movements in increasingly intentional and goal-directed ways (Thelen and Smith, 1994), and of the profound ways that a lack of predictable movements and reflexes would alter that dynamic, creating a developmental cascade that flows with increasing velocity toward an autism diagnosis (Maurer and Damasio, 1982). A related observation made by viewers of the video clips is that they are witnessing the unfolding of adaptive developmental *differences* rather than a display of static deficits. These children are co-adapting with their environment, determinedly brokering complex and probably exhausting deals with their bodies in order to keep moving and exploring. The video vignettes tell a very different story from the presumption of autistic indifference, and refute the conflation of movement challenges with lesser intelligence, “task avoidance,” or the desire to self-injure or aggress. Witnessed at an early age, without judgmental interpretations, it is easier to confirm the words of self-advocates such as Tom Page, who states: “*My senses and body parts did not work as a unit*” (Young, 2011, p. 166).

## Dyssynchrony and subroutines

These observations of early development fit well with data from a variety of fields, including neuropsychology, neurophysiology, and neuroimaging, which suggest that autism is fundamentally a “Temporo-Spatial Processing Disorder (TSPD) of multi-sensory flows”:

TSPDs include various degrees of disability in i) processing multi-sensory stimuli online, ii) associating them into meaningful and coherent patterns and iii) producing real-time sensory-motor adjustments and motor outputs (Gepner and Féron, 2009, abstract).

TSPDs, defined to include a range of conditions from attention deficit disorder and dyslexia to Parkinson's disease, reflect “disconnectivity” or “dyssynchrony” across multiple neurofunctional systems and would be expected to play out in the realm of “perceiving, imitating, understanding and producing emotional and verbal events on time, and therefore in interacting here and now with (the) human and social environment” (2009, p 1238).

These predictable expressions of connectivity challenges parallel, and may help explain, the diagnostic “autism triad” centering on impairments in communication, impairments in social interaction, and restrictive interests and repetitive behavior (American Psychiatric Association, 2000). Communication and social interaction are highly sensitive to mis-timing; an appearance of impairment may arise from processing demands the system cannot meet. Repetition or restriction of experiences may represent an actual adaptation to dyssynchrony: if you cannot slow the pace of demands, at least limit their number. Significantly, the proposed effects of TSPDs are reflected in the self-reports of people diagnosed with autism. As Tom Page recalls:

*In the beginning of my life, I was a frightened little boy. I remember being confused most of the time. People were doing things for no reason that I could make out. I seemed to be doing things for no reason they could make out. Neither could understand each other's actions. Their mouths moved and made sounds that made little sense to me* (Young, 2011, p. 76).

Page and many others relate experiences of profound dyssynchrony, in which the different sensory aspects of a situation fail to match up coherently; ultimately, intentions and actions may themselves be uncoupled. This may occur temporarily and without warning as automatic movement “subroutines” cease to function as team players and emerge into prominence on their own. Subroutines are fixed action patterns that we all rely on, and don't notice as long as they are working in sync under the auspices of our general intentions (MacLean, 1990). We stand up “automatically” when called upon; appropriately produce a social smile; or effortlessly turn a corner as we continue to walk onward, uniting two action patterns in a superimposed movement (which children in the Teitelbaum et al. (1998) and Teitelbaum et al. (2004) videos were unable to effect). We don't have to plan these subroutines consciously because they are triggered by and subservient to the larger scheme of action in which we are involved, and that scheme in turn is coupled or entrained to social and environmental cues.

But for people with autism (and other familiar conditions, such as traumatic brain injury and Parkinson's) these fixed action patterns can take on a life of their own that may look—and feel to the person—confusing and even alien, as if they were coming from somewhere else. My own daughter, taken to task for her wandering ways when she repeatedly left her elementary school classroom, put her case succinctly: “*My brain doesn't tell my legs what to do; my legs tell my brain what to do*.” Several of my adult acquaintances with autism will unexpectedly reach out to touch objects while disavowing any interest in doing so. Nor is this phenomenon limited to the gross motor domain; for example, Sue Rubin warns others to attend to her typed communication and not necessarily to her speech, which may be unrelated (Biklen et al., 2005, pp. 92–93). Barb Rentenbach cautions, “*My facial expressions don't always match my emotions* (Young, 2011, p. 163), and indeed many people with autism are faulted for displaying the “wrong” affect, which may be interpreted by others as signifying insensitivity. Nick Pentzell, a gifted college scholar, writes candidly of disagreeing with his body's activities:

*I tell (my body) to go to sleep, but it leaps on my bed. I tell it to want good and it goes for bad. I open the door to maturity and it slams it in my face* (Young, 2011, p.163).

Similar reflections account for a large portion of the autobiographical autism literature. A person whose body is running competing subroutines is easily misperceived as failing to employ willpower. Yet as Barbara Moran memorably put it:

*If only people knew the reason why autistic people get upset so easily. Self-control is much harder because there is so much “self” to control* (Autism Support and Advocacy in Pennsylvania, n.d.).

Overstimulation of a delicately-balanced sensorimotor system appears to be a frequent factor in this uncoupling of intention and action (Markram et al., 2007). The peripheral nervous systems are involved: stress is placed on the autonomic systems that control visceral responses, and in particular on the sympathetic and parasympathetic systems, which must function in a complementary manner to regulate physiological responses and ultimately social behavior (Porges, 2003). As the balance between arousal and inhibition goes awry, the results are unintentional and unanticipated. This unpredictability of how and when one's body will lose balance is another frequent theme in the autobiographical literature of autism. As Sue Rubin observes:

*Autism plays on a person's five senses. It can vary from day to day and is not something one can control or see coming* (Biklen et al., 2005, p. 103).

For “neurotypical” individuals who take their neurosynchrony for granted, it can be difficult to envision what transient connectivity challenges would feel like. One useful image might be the croquet game in Lewis Carroll's *Alice's Adventures in Wonderland*: (Carroll, 2002, orig. 1866) when the components of the game (i.e., flamingo for the mallet, hedgehog for the ball, the Queen's guards for hoops) are pursuing their own subroutines, the game is impossible to play. Despite her knowledge of the rules, at those times Alice will be mocked as incompetent. But we also know that there will be fortunate moments when all systems are in sync and a structured, non-chaotic game will emerge. Similarly, parents and teachers report incidents when people with autism surpass their typical level of performance with a virtuoso display, such as the “non-speaking” individual who suddenly makes a highly articulate statement, only to lapse back into silence, or the person who executes a perfectly coordinated gymnastic move once every few years. These may be occasions when a delicately balanced sensorimotor system momentarily achieves full harmony.

The existence of this non-volitional performance variability may encourage us to wonder about the ways in which typical education and treatment goals for people with autism are structured, with success defined and knowledge measured in predictable repetitions of a task (e.g., 9 times out of 10, with 90% accuracy), and with connectivity further destabilized by mounting pressure, time constraints, and increased demands to repeat a successful act. If these speculations about neural connectivity are correct, we inadvertently may be creating the very types of expectations and circumstances most likely to frustrate performance and to lead to underestimations of knowledge.

People with Parkinson's and related conditions may devise ways to trigger missing subroutines (such as those that help initiate walking), or to re-integrate and utilize intentionally movement patterns that have gone astray, by means of movement and rhythm accommodations (Sacks, 1990). The triggering of allied reflexes—using intact movement patterns to indirectly initiate the desired but inaccessible movement—is a potentially useful strategy. Some approaches to autism support attempts to restart or reintegrate movement through similar accommodations, including modeling the action to be performed; moving with the person; using indirection to trigger a recalcitrant movement; enhancing proprioception via touch, deep pressure, or rhythm; and incorporating subroutines via the Rapid Prompting Method (Chen et al., 2012).

## Timing and the binding window

In autism, the typical rhythms of sensory and social connectivity may be disrupted in a number of ways, only a few of which have begun to be investigated in any depth. Starting in the 1960's, William Condon looked at the importance of self-synchrony (the effective coordination of one's own body) and interactional synchrony (coordination of one's own movements with those of others) for communication and social interaction. He suggested that these core processes were challenging for people with autism because sound processing was both delayed and triggered multiple responses, as if a sound were echoing. Condon found that children with autism would “entrain” or respond to the sound first on their left side, followed by a delayed response on the right (the opposite pattern occurred with children who had dyslexia) (Condon, 1974, 1975, 1985).

Condon theorized that these disruptions would compromise the crucial sharing of experiences from an early stage of development, causing the closely-timed, rhythmic interactions between child and caregiver—and the unified audiovisual experiences they create—to falter (Condon, 1979). Such babies would appear highly distracted; due to mistiming, they would perceive their sensory world to lack pattern and focus.

Condon became interested in the use of carefully attuned rhythm-based interventions in helping to support both self-synchrony and interactional synchrony; the film “Looking for Me,” (1970) in which dance teacher Janet Adler works to communicate at the body level with two young children with autism, grew out of one of his projects of that era. These same concerns about how to conduct a dance of relationship with children on the autism spectrum reemerged in the Developmental, Individual-difference, Relationship-based (DIR) approach which took shape during the 1980's and came to prominence in the 1990's (Greenspan, 1992, 1997; Greenspan and Wieder, 1997, 2006).

While this early work suggested that registering and responding to sounds is not a tight, efficient process for people with autism, recent research on audiovisual processing has found that the “binding window”—the window of time in which the input from different sensory modes occurs closely enough to ascribe it to the same event—was twice as long for subjects with autism as for the control group (Foss-Feig et al., 2010). The times involved may seem vanishingly small—600 ms vs. 300 ms, respectively—but at the neurological level that can be enough to inhibit or prevent multisensory experiences from binding into a single well-integrated perception. Sights, sounds, and perhaps other sensory information, would not match up smoothly; unrelated events might be perceived as connected, while aspects of the same event might be experienced without the precise timing (e.g., of speech sound and facial movement) that creates meaning. This would leave the person straining for coherence and, perhaps, adapting by trying to limit input coming through the “window” to one perceptual mode at a time. Foss-Feig and colleagues suggest possible outcomes of a wide binding window, including interferences in responding to input, difficulty identifying the source modality of input, and changes in information content sufficient to “endow social interaction with confusing and irrelevant associations” (2010, pp. 387–388).

The biographical autism literature may offer instances of “confusing and irrelevant associations” that have become locked in, to the detriment of social interaction. For example, Sean Barron, who with his mother wrote *There*'*s a Boy in Here*, vividly recalls his anger when bus 24 at his elementary school arrived late, depriving him of the pleasure of seeing the entire fleet lined up (Barron and Barron, 1992, p. 108). So strong was this initial association of the number 24 with disappointment that over time it attached to other things designated with that number (e.g., marbles and playing cards that he felt compelled to purge), eventually including a teacher whose friendly overtures he repeatedly rejected upon learning that she was 24 years old (Barron and Barron, 1992, pp. 151–152).

Linking his unhappy experience with bus 24 with his teacher's age was unfortunate for Sean, since it removed the possibility of getting to know her as more than a number. But seeing such activities not as examples of irrational or asocial behavior, but as emerging from an active process of trying to associate perceptions—perhaps in the presence of an extended binding window that opens upon an overly generous array of possibilities—profoundly changes the usual autism narrative. There is also no reason why unusual perceptual associations need be detrimental; some may reveal interesting perspectives and creative possibilities, as suggested in the anecdotal literature about the prevalence of synesthesia on the autism spectrum and the pleasurable, imaginative uses to which such associations may be put (Tammet, 2006).

## Multi-modal/cross-modal coordination

Research on typical infant development offers important clues to what may be happening when people with autism try to assemble or bind coherent multi-sensory experiences. Called multi-modal, cross-modal, inter-sensory, or multi-channel coordination, it involves the crucial ability to create a unified whole out of perceptions from different sensory channels, as when a baby registers visual recognition of an object she previously has only explored by touch, or recognizes that a certain sound is associated with his cup hitting the floor rather than with other nearby events. Infants are born ready to start building stable multi-modal perceptions out of sensory stimuli; it is this emergent capacity that keeps their experiences from being, in the oft-quoted words of William James, “one great blooming, buzzing confusion” (1890, p. 462).

The question is how infants, or any of us, take the multi-modal stimuli arriving through different senses and construct a unitary event. The answer appears to be that infants make use of *amodal* stimulation that cuts across the boundaries of different sensory modes:

Amodal information is information, such as synchrony, rhythm, tempo, and intensity, that can be detected in more than one sense modality. Detecting this information promotes the processing of unitary multimodal events in young infants (Bahrick and Lickliter, 2004, p. 137).

Amodal properties may act as universal attractors that pull diverse sensory input into recognizable patterns. When the amodal information perceived though one sensory channel is also perceived through another channel, a match is made, supporting the unification of both streams of information into a seamless whole. Bahrick and Lickliter cite extensive research demonstrating the use of amodal information by infants to link the experience of faces and voices, and specifically of lip movements with speech; to detect visual and auditory indicators of emotion; and to “match objects and sounds on the basis of temporal synchrony, tempo, rhythm, and temporal microstructure specifying the substance and composition of objects” (2004, p. 137).

This research on how typically-developing infants bind sensory perceptions references the very types of experiences with which infants and children with autism are known to struggle. It seems possible that, if the detection of amodal properties that should unite such basic sensory experiences were perturbed by mis-timing, the experience of synchrony that allows even arbitrary, socially-mediated relations, such as that between an object and a speech sound, to be detected by an infant (Gogate and Bahrick, 1998) would be inhibited (Guiraud et al., 2012). The difficulty experienced by children with autism in constructing coherent perceptions of basic but multimodal social and emotional cues could be a predictable outcome of a wide binding window that leaves the different sensory aspects of an event confusingly out of sync. The frequent preference many of these children demonstrate for unimodal stimuli—for exploring the world one sensory channel at a time (Grandin and Scariano, 1986; Grandin, 1995, 2000)—may constitute a reasonable alternative strategy, and tend to result over time in increasingly different ways of organizing attention and perceptual categories (Marco et al., 2011).

Our ability to synchronize stimulation from different sensory modes into a coherent experience is not just a curiosity of brain science; it reveals a process vital to cognition. In their groundbreaking work on infant development as a dynamic and emergent, rather than pre-scripted, process, Thelen and Smith review the research on cross-modal or intersensory performance and suggest:

…the developmental significance may be far more than that intersensory coordination exists. Indeed, we believe that what we are observing in experiments is the very mechanism of development—not a product, but the process through which intelligent commerce with the world is selected and maintained. In our view, what experimental tests of cross-model performance do is reveal how perception-action categories—the fundamental stuff of cognitive development—are selected in real time (Thelen and Smith, 1994, p. 192).

Thelen, Smith, and colleagues used the principles of dynamic systems theory to explore how sensorimotor systems, thought processes, and the self develop through ongoing entrainment with the physical and social environments. If development is an ongoing process of coordination through which body and environment are mutually shaped and explored, then the not-yet-diagnosed babies in the home videos studied by Teitelbaum et al. (1998) and Teitelbaum et al. (2004) are caught in the act of developing and creating their worlds, not of demonstrating deficits. People with autism are, of course, continually perceiving and moving, just as they are undertaking “intelligent commerce with the world” as they continue to evaluate and revise their perceptual categories. But, to quote the memorable title of Sue Rubin's 2004 documentary about her life, “*Autism is a World*”; intelligent commerce with that world may need to recognize a different currency and the existence of a different economy.

That economy may turn out to be based less on the real time coordination of the different currencies represented by our different sensory modes, than on the opportunity to pay them out one at a time, at different rates and over different time periods. Williams (1992, 1994, 1996, 1998) and other self-advocates have recounted their challenges in processing multimodal stimuli, especially for long periods or when trying to assimilate new information. Alberto Frugone writes of his difficulty processing auditory and visual information simultaneously:

*For example, I'm sitting in front of the TV set, I hear the words and I can decipher their meaning, but I don't use my visual perception simultaneously, otherwise my attention would go* (Biklen et al., 2005, p. 196).

My second son found his own solution to this problem in the close captioning feature of the TV, which he used to reduce stimulation to one (visual) mode. My daughter took the opposite approach, “watching” her favorite cartoon show by retreating to the upstairs hallway so that only the distant sound was available. To prevent processing overload, my oldest son's conversational rhythm involves frequent pacing in and out of the room. Given a computer, he immediately developed a preference for emailing—even with people under the same roof. The ability to organize communication according to his own rhythms pays great dividends: his typed conversations are long and eloquent, in contrast to the more cursory messages of his “live” conversation.

A preference for uni-modal and highly systematized patterns of exploration is common on the autism spectrum, and may represent an accommodation to sensory differences. *There*'*s a Boy in Here* chronicles episodes that may suggest how the young Sean worked to piece together the developmental experiences he needed, in the face of frustrating connectivity challenges. For example, Sean recollects how, as a preschooler, he was engrossed in certain types of activities:

*I got enormous pleasure from throwing things into a big tree in our backyard. It didn't matter to me what shape or size the object was—I took toys out of the sandbox or things from the kitchen … I wanted to see how high they would go and where they would get caught. I loved the pattern: throwing the object as high as I could, seeing where it hit the tree, following its downward movement with my eyes, and watching where it got stuck* (Barron and Barron, 1992, p. 44).

Years later Sean's investigations expanded beyond his backyard:

*I had an intense interest in dead-end streets. The things I liked to do, in general, were those that offered some variation but were still repetitious. So dead-end streets were perfect. I knew the different ways that such streets could look. Two neighboring streets could both be dead-ends but look and feel totally unlike each other. Yet they both ended, and in that way they were the same* (Barron and Barron, 1992, p. 89).

It would be easy to label this behavior as “perseverative” or to classify it as a sign of intellectual disability, but that would not respond to Sean's obvious intelligence or to his memory of actively experimenting with patterns and categories. Other self-advocates have reported similar motivations for similar activities:

*The inability to get consistent meaning through any of my senses in an environment that demanded that I did, meant that I developed another side; a side with an acute ability to respond, not to meaning but to patterns* (Williams, 1996, p. 242).

Such observations might encourage us to ask: Was Sean trying to establish satisfying patterns among various perceptions in the face of difficulties (such as an enlarged binding window) that made each experience potentially novel and challenging to align and compare? Did the controlled variations in dead-end streets attract exploration for similar reasons?

It seems possible that some of the play strategies of children with autism, many of which involve the exploration of small differences introduced into repeated enactments of an established pattern, may represent adaptations to sensory processing challenges and attempts to overcome them by self-imposing a rhythm, pace, and finely-detailed scale acceptable to the demands of their sensorimotor systems. Seeking and systematizing fundamental patterns may be an intelligent and sensible strategy if experience is often overwhelming and refractory at the perceptual level. In the absence of a reliable sense of embodied movement through space and time, these repeated patterns may provide a frame of reference—a sort of prosthesis for the nervous systems. So far, however, exploratory play on the autism spectrum remains understudied and is discounted by some as a negligible domain. These possibilities are raised in a spirit of humility, because we have known so little (and have been content to assume there was so little to know) about how children and adults with autism explore their environments, and about the timeframes and rhythms through which their movement and perceptual systems operate.

## A sense of time

It is often observed that the sense of time appears to work differently for many people with autism. That would not be surprising, given the increasing evidence that autism involves challenges to neural connectivity and different ways of assembling experiences. What has to be connected in order to accurately sense time is something even more complicated than, for example, connecting speech sounds with facial movements. Time is not a mode or channel of sensory experience, but an amodal property that unites the perceptions of different senses. We sense time through comparisons of our experiences, bootstrapping from events of known duration to establish expectations about other events; repeated events in the world and familiar rhythms of the body come to stand for intervals of time, with which new events can be compared (Lakoff and Johnson, 1999, pp. 128–139).

If these embodied experiences are unreliable for people on the autism spectrum, it might make sense that the comparison process also would prove challenging, resulting in a panicked feeling of being adrift in a sea of time. Preliminary research has suggested that development of an accurate perception of temporal duration may be delayed among people with autism, and that this delay may help explain certain key diagnostic features (Allman, 2011; Allman et al., 2011). It seems possible that the deep satisfaction many people with autism find in repetitious activities—such as my oldest son's strong inclination, in early childhood, to repeatedly turn lights on and off—may have something to do with a need to ingrain the experience of these temporal units by participating in a pattern as replicable and predictable as a pendulum or metronome. Oliver Sacks has noted that patients with Parkinson's disease (a movement disorder with certain similarities to autism, including slowed gait and speech, and difficulty initiating actions) can be “activated and regulated, ordered and organized” by measures such as

…stairs, steps painted on the ground, clocks, metronomes, and devices that count in a simple, regular, and orderly manner; or by co-action and co-ordination with a concrete, living activity or agent (1990, p. 347).

It remains to be seen whether and how people with autism might be inventing similar mechanisms to self-regulate and, if so, how the possibility of co-action with other persons—skilled partners in the dance of relationship—might be more deftly exploited to enhance temporal awareness and praxis.

Sensing time with reasonable accuracy has enormous consequences for anticipating, planning, inhibiting unwanted responses, and mitigating anxiety, which flourishes when expectations are violated or not established. Caregivers often note the rising panic of a child with autism who faces a non-preferred task and seems unable to call upon any guiding sense of when or whether it will end. Similarly, a person with autism may be unable to be guided by a sense of time in anticipating desirable events. In the book *Strange Son*, many of Tito Mukhopadhyay's challenges are described in terms of his difficult relationship with time:

He did not experience time the way most people did… He was anxious all the time because he could not anticipate what was next. When (his mother) told him anything having to do with future events, his anxiety redoubled because he could not tolerate the thought of getting from the present moment to a designated time in the future. If he wanted something, he had to have it right now (Iversen, 2006, p. 143).

Time-based challenges to perception and action may turn out to be highly varied, involving not only time future but time present. Leary and Hill (1996) compiled descriptions of many movement differences in autism that may present as difficulties in timing, including instances of individuals becoming stuck in an activity (time never stops), or frozen in catatonic states (time never starts). Damasio and Maurer (1978), Vilensky et al. (1981) identified Parkinsonian symptoms in the gait of people with autism that involved moving to a slower internal clock, and more recent studies have confirmed related challenges manifested in arm movements (Mari et al., 2003). Respecting, and not disrupting, that internal clock can be a powerful accommodation. During my oldest son's adolescence, when his self-regulation seemed especially challenged, I was frequently baffled by his tendency to “freeze” in place for extended periods just as we were running late. I queried Ralph Maurer, a psychiatrist and director of a university-based autism center, who suggested that these abrupt stops, usually followed by a period of rapidly shaking a string or fingers near his eyes, may be my son's way of resetting his internal clock when my fast pace had jammed the gears. (Many people with autism describe the latter activity as a way to slow the demands of perception by segmenting it into still frames, like viewing a “flip book” animation). Considered from this angle, the situation improved enormously when I slowed down or waited silently, so that my rhythms would not overwhelm his own.

## Timing and emotion

Timing our actions to accord with the actions of others is vital to our experience of emotion, and the success or failure of mutual timing can profoundly influence our relationships with and feelings about others. An out of sync phone conversation, with both parties repeatedly talking at once, will probably end in negative feelings; movies in which the sound is out of sync with the actors' lips prevent us from engaging emotionally. Research into the Social Engagement System of people with autism and related conditions suggests that dyssynchronies of the autonomic nervous system are deeply implicated in the kinds of timing breakdowns that can subvert the dance of relationship and emotional development (Porges, 2003). Facial expression, head gesture, the ability to rapidly extract speech sounds from ambient noise, the prosody or rhythm and timing of spoken language, and social interaction in general can be compromised; as the phylogenetically more recent and more directly socially-mediated mechanisms falter, the nervous system reorganizes around “the adaptive defensive strategies of mobilization (i.e., fight or flight behaviors) or immobilization (i.e., shutdown)” (Porges, 2003, p. 508). These non-volitional responses are frequently observed among people diagnosed with autism. Since the peripheral and central nervous systems are bidirectional and intertwined, they may both exacerbate and reflect the dysregulated cardiopulmonary and digestive activities found on the autism spectrum, inhibiting entrainment with the circadian rhythms around which key aspects of social life are organized.

Successful social engagement therefore may need to be approached as an alert, intentional process that is deeply embodied and meets the person with autism on their own terms, avoiding the triggering of defenses. If emotional and social communication is to occur, it cannot be a disembodied one-way process like feeding data into a computer; participants must rise to the challenge of co-creating a synchronous experience (De Jaegher, 2006, p. 186). Sean Barron offers a rare inside view of what a successfully coordinated interaction could feel like to a person who has seldom been able to sustain one. He recounts how, after his family relocated to California, he entered a new high school. His school experiences had previously been confusing and lonely, and his expectations were low as his sister Megan introduced him to her new friend Dianne:

*Megan and Dianne went to sit down under a large tree, and I stood where I was. Meg looked back at me. “Sean, come and join us!” I did. Several other kids came over and sat down, and I was introduced to all of them. Everyone chatted about school, but I couldn't really hear them—there was a kind of hum inside me that I later realized was happiness. I was very aware that as they talked, they looked at me, too, that they were including me in their group. I believe I'll never forget that day ….Sitting under that tree, I had the first relaxed moments of my life. I began to feel safe enough to listen to the other kids, and the amazing thing was that I understood what they were saying! It all made sense to me* (Barron and Barron, 1992, p. 218).

It seems significant that Sean's surprise over feeling safe and relaxed, followed by his sudden realization that he can access the emotion and understand the communication taking place, matches the prediction that “the perception of safety is the primary requirement” in successful intervention, since it prevents “degrading of the function of the Social Engagement System” (Porges, 2003, p. 511). Given a safe and respectful setting in which to organize his perceptions, Sean displays intelligent and highly motivated efforts to piece together experiences that did not immediately present in a unified way: the hum inside and the outer event, leading to the dawning realization that this is what is meant by happiness. We note the time required (in both minutes of real time and years of developmental time) for him to make sense of the unfolding situation. Above all, we note the dance of relationship that takes place, and the “meaningfulness” that it supports (De Jaegher, 2013).

The ability and determination to connect experiences and probe unexpected similarities also drives creativity, art, imagination, and insight; it makes humans unpredictable and sometimes mistaken, but more than just automata. By now the phenomenon of people with autism, including the most severe challenges, excelling in various creative endeavors has become almost a commonplace; from Tito Mukhopadhyay's heartfelt poetry to Larry Bissonnette's (Biklen et al., 2005) witty and allusive paintings, we recognize that people with autism are interested in, and able to create, new and unexpected conceptual linkages out of the raw stuff of experience.

The formation of, and often intense emotional investment in, unusual categories of things by people on the spectrum might also be explicable as a tendency of this developmental difference to support a wide variety of unusual, creative associations (including complex algorithms for calculating and recalling them). Referred to as “preferred interests” or “passions,” and sometimes rising to the level of “savant skills,” they can be a motivating force that powers development if approached respectfully. Even an enthusiasm which at first glance seems narrow can ultimately be linked to a potentially limitless array of other topics. From the time he was a toddler, my oldest son was fascinated with big industrial storage tanks. While this was not a category of object that appealed to most children, he experienced them as awe-inspiring. We took trips to admire storage tanks the way others travel to view the Pyramids. Examining them visually may have served an exploratory function similar to the play with buckets and boxes through which his age peers developed the concept of containment (Lakoff and Johnson, 1980, pp. 30–32), but on a more heroic scale. We engaged with him around this interest, eventually introducing him to laboratory beakers, which were “like objects” that also stored chemicals in rounded containers. When, in adolescence, he made the leap from beakers to an interest in test tubes, we began to glimpse a career; as an adult, he is now employed as a phlebotomy technician, enthusiastically filling test tubes at a local hospital.

Engaging in these intense interests with a person with autism can be the first step in a dance of relationship that introduces us to the world they perceive, and allows us to become more in sync with their rhythms of exploration and development (Stillman, 2009, pp. 139–147). Objects and activities to which a person with autism gravitates may turn out to be no stranger than the typical objects and “rituals” that most people use to reassure themselves that the world is orderly, knowable, and meaningful. The impetus to find it so seems universal. That person who looks disconnected and out of step may, for all we know, be engaged in a deeply felt activity that evokes an experience of transcendent connectivity and harmony. As Sue Rubin explains:

*As someone would carry around a lucky coin or rabbit's foot, I tend to walk about with a plastic item such as a spoon or plastic button in hand ….Water, in which I also find great comfort and joy, is something that falls with an unexplainable grace. For that split second that the water falls, I can almost see into another world* (Biklen et al., 2005, pp. 83–84).

## Suggestions for research and practice

These observations about rhythm and timing are not intended to suggest yet another thing that is “wrong with” people diagnosed with autism. Nor are they intended to provide a new set of instant explanations for why a particular person does certain particular things. As Douglas Biklen reminds us,

The traverse from neurology to behavior is a remarkably elusive one, yet the tendency to treat it as direct, obvious, and specific can occur without hesitation (Biklen et al., 2005, p. 35).

Perhaps bringing rhythm and timing into our field of vision *will* cause us to hesitate, and orient us in a better direction: away from static depictions of behavior as discrete items with firm boundaries, and away from the kind of indiscriminate reductionism that requires the sacrifice of more dynamic questions and observations. Colwyn Trevarthen speaks for and with a growing cohort of researchers in lamenting that in much of current psychology

Neither the purposes of the individual human being, nor the meaning built by sharing of purposes, experiences and feelings between consciously active and mutually aware subjects, are explained (Gallese and Lakoff, 2005) (Trevarthen, 2011, p. 122).

When we consider rhythm and timing, the significance of evolving relationships, of personal history, and of the process of striving for meaning, come back into focus. We start to recognize the ways people respond to sensorimotor obstacles by negotiating hard-won and fragile internal treaties that allow them to keep operating. We give ourselves permission to think about how people develop, moment by moment, as the sum of their entire, irreducible history of embodied perceptual experiences. We listen seriously to self-advocates like Barbara Moran, who assures us, “*My mind gets there in the end; but it takes the scenic route*” (Donnellan and Leary, 1995, p. 45).

Clearly there is a huge impact on development when a child's perceptual experiences are out of sync and he or she struggles with challenges to bodily and social connectivity. Attending to rhythm and timing may offer new, more insightful ways to respond. With such a goal in mind, here are some possible directions we could explore more thoroughly, both as everyday practitioners and as researchers:

1. Bring relationships and their development to the forefront of our work; emphasize reciprocal relationships in which both partners give and take. Reciprocity is not the same as teaching, training, modifying behavior, overseeing a child's play, or general caretaking. It should be understood as an intentional, active process of sharing the child's world, one in which we “need to become something of a detective to discern the ways that the child is expressing joint attention and social and emotional reciprocity” (Gernsbacher, 2006, p. 145). Ralph Maurer suggests that if we commit ourselves to learn how relationship works we might discover “a missing behavior technology ….that uses concurrent stimuli to exploit oscillators”—in other words, one with the power to “compensate for the children's deficiencies in that dance of relationship, like Arthur Murray [dance] instructors, and then work from within the dance to expand the child's world, like mothers do with infants” (Maurer, 1995, p. 2).
2. Work with, not on, people with autism (Lovett, 1996); support them to explore their preferred interests and to expand them in directions that others can share, rather than controlling access to these interests and exploiting them contingently. Remember that the things that motivate and make sense to people with autism can become the foundation for their explorations of the larger environment. Researchers might consider the complex connections between sensory and motor challenges and the emergence of particular kinds of experiential categories as key features of a child's motivational landscape, and seek ways to engage more substantially around a child's interests and support their elaboration and connection with categories that are meaningful to caregivers, peers, and the child's culture.
3. Avoid the tendency to concentrate on abstractions at the expense of real life experiences (e.g., memorizing rote “facts” without seeking ways to apply them; learning to identify pictures of activities rather than engaging in them) or to create simulated, out-of-context experiences (e.g., token economies, “pretend” shopping, “job-like settings” with pointless tasks). Self-advocate Alberto Frugone puts it this way:
  *It's necessary for me to gain real experience. While trying to perform an action, even if my gestures are difficult, I obtain valid practice. But it has to be a practical, contextual action not an artificial situation* (Biklen et al., 2005, p. 187).
  Lack of access to meaningful, typical experiences may result in knowledge gaps that lead to low appraisals of a person's intelligence and to stigmatization. Now that decades of research and practice have assured us that discrete skills rehearsed in isolation do not tend to generalize well (Koegel and Koegel, 1996, 2006), researchers might seek new ways to support “valid practice” while avoiding the perils of prompt dependence and unnecessarily intrusive physical support.
4. Value exploration over replication as new activities are learned and transitions are negotiated. It is possible to place too high a priority on having a person with autism do things the same way and follow the same routine every time. Our growing understanding of dynamic systems suggests that encouraging flexibility and supporting a person to experiment with different solutions to a task may be crucial for successful adaptation. Researchers may wish to reconsider their data collection to incorporate variability itself (Thelen and Smith, 1994, pp. 86–88), not as randomness or noise in the system (or, in the diagnostic terms of autism, self-stimulation or off-task behavior) but as developmental data worthy of closer attention.
5. Slow down; work and communicate at a longer, slower rhythm. Give longer wait times to allow the person to process meaning and formulate a response. Create safety; reduce anxiety through techniques that relax body and mind, such as deep breathing, yoga, and “mindfulness” (Kabat-Zinn, 1991). Many parents and therapists successfully utilize music, rhythm, and dance to support and explore emotions and scaffold communication. Researchers have found useful therapeutic models in the coordination of body rhythms between typically developing infants and caregivers, and could explore new ways to adapt them to infants and children whose sensorimotor systems may not be disposed to find that early social dance coherent or compelling. as well as to older individuals for whom the dance faltered at an early stage.
6. Try communicating via a single sensory channel or mode at a time; minimize multisensory stimulation, especially when teaching something new, or when a person is tired or stressed. Consider whether multimodal goals, such as making eye contact while conversing, are furthering or frustrating comprehension and performance. Given that proprioceptive feedback for many people with autism may be inadequate, researchers might consider whether it is possible to devise more salient ways to present and guide proprioceptive experiences, such as by re-routing them through a preferred perceptual channel (e.g., so that body location and position could be experienced through sounds, lights, or haptic feedback triggered by movement).
7. Create accommodations for sensory and movement differences. Since these differences are generally not under a person's direct control and don't respond well to demand situations, we can respond instead by supporting the person to “work around” these challenges via personalized solutions (Donnellan et al., 2010; Leary and Donnellan, 2012), and by exploring environmental, interactional, and self-regulatory adjustments that enhance praxis. It may be useful to examine the supports and accommodations that are known to work for people with neurologically similar experiences, such as the challenges to gait and timing in Parkinson's, to determine whether they can be successfully adapted to support timely initiation and enhanced coordination for people with autism.
8. Assume that the person on the autism spectrum is intelligent, has the capacity to learn, and is motivated to make sense of his or her experiences. Make decisions based on the criterion of the least dangerous assumption, which states that:
  …in the absence of conclusive data, educational decisions ought to be based on assumptions which, if incorrect, will have the least dangerous effect on the likelihood that students will be able to function independently as adults (Donnellan, 1984, p. 141)
  and that
  …there is less danger to students if teachers assume that poor performance is due to instructional inadequacy rather than to student deficits (Donnellan, 1984, p. 147).
  The implications of this principle for research may prove to be profound: with the connectivity research suggesting that performance among people with autism is highly sensitive to internal and external conditions, and easily disrupted, research design deserves increased scrutiny. Factors that were once considered to have no impact, or to be cleanly separable from the experimental situation, may have to be reconsidered. The results of some past experiments may become open to reinterpretation—possibly in very exciting and productive ways—based on new questions about task design and presentation.
9. Explore schedules, checklists, images and pictures, flow charts, and timelines; clocks and timers with visual representations and sound cues; the use of songs, melodies, or simple beats to establish a predictable rhythm and timeframe; and similar customized strategies and devices that appeal to different senses to make the passage of time easier to experience and to track. The importance of structuring tasks and information clearly, assuring that essential features are salient and minimizing sensory and conceptual clutter, is widely appreciated. These features of task design appear to compensate for difficulties with rhythm and timing, but are under-researched and in need of experimental refinement.
10. Consider that some people may be more talented than others at finding and matching the rhythms of people with autism. Training can help, but not always. The presence or absence of this ability may be a non-trivial factor in providing successful support. The support of a sensitive “dance” partner may also turn out to be the active ingredient that explains the efficacy of certain methods and approaches “for autism” which otherwise defy explanation. It would be helpful to reevaluate puzzling or inconclusive data on treatment efficacy, particularly from studies that posited a significant placebo effect, with an eye toward analyzing the movement, rhythm, timing, and overall impact of the person(s) partnering or interacting with the subject(s) with autism; they may be, or be supplying, the active ingredient that is driving the change.

## On research focus and design

In summary, this parent proposes that it is time to take a break from the enumeration of what people with autism appear to be “not doing” and construct a research agenda based on the assumption that they *are* exploring and developing, and that investigating *how* that is occurring will open new vistas. If any area of study can force us to leave teleology at the door, as the price of admission, it is autism. Measured as progress toward pre-defined and self-obvious goals, development in autism becomes a dry account of missed marks; when activity and adaptation are given primacy in research and practice, we begin to see differently.

What we are seeing is a developmental difference that appears to be marked by profound challenges to neurological connectivity, resulting in a cascade of confusing perceptual experiences that disrupts the finely-tuned choreography of social interaction. A promising question researchers might ask concerns the role of rhythm and timing in the rapid, yet highly sensitive, operations involved in piecing together coherent sensory and motor experience, and whether temporal accommodations and supports can be mobilized to reduce an overloaded processing system and enhance performance. Is there plasticity in the perceptual and motor systems of children diagnosed with autism, and does it differ in speed and degree according to type of sensory input, task structure, and the type of accommodations and supports utilized to guide them?

Evidence is mounting that this may be so: for example, research on Musical Interaction Therapy suggests interventions that can be used to overcome social timing challenges and build a scaffold for the emergence of communication and language (Wimpory and Nash, 1999; Wimpory et al., 2007); similar work is being done through Neurologic Music Therapy by practitioners such as Hardy (Hardy and LaGasse, 2013). An ongoing study by neuroscientist Elizabeth Torres is developing computer-based supports that may assist children with autism to cope with the randomness and noisiness of their actions, which seem to involve a reduced distinction between intentional and unintentional movement (DeWeerdt, 2012). Documenting such plasticity, and identifying the types of supports and accommodations to which it responds, would be a significant step toward improving praxis so that people with autism can more effectively realize their potential.

In researching performances that are highly sensitive to many variables, we must face an issue that many autism researchers have so far been content to set aside: accountability for the impact of researchers themselves on the test situation, including their place in a complex history of beliefs and assumptions about autism, and how these might impact their ability to design and engage subjects in meaningful test protocols. A lab coat is not a Harry Potter-style cloak of invisibility, and it is only the now-fading presumption that people with autism operate independently of and indifferently to the environment and social world that has allowed much research to go forth without addressing such issues. A dedicated research focus on what individuals with autism actually experience, what they intend and attempt to do (and how this happens in the context of movements their bodies inadvertently produce), how they play and explore, and the accommodations and supports they require to make sense of daily life, may prove enlightening (Robledo et al., 2012), and encourage us to include people with autism (and their families) in designing future research. There is much to be said for self-advocates' concept of autism not as a pathology but as a culture or way of perceiving—as Sue Rubin says, “*a world*”—and the way to approach a culture or world is to engage with it open-mindedly, in the spirit of harmonizing with the rhythms of a different drummer and “learning to dance.”

### Conflict of interest statement

The author declares that the research was conducted in the absence of any commercial or financial relationships that could be construed as a potential conflict of interest.
